# Charge Carrier Diffusion Dynamics in Multisized Quaternary Alkylammonium-Capped CsPbBr_3_ Perovskite Nanocrystal Solids

**DOI:** 10.1021/acsami.1c11676

**Published:** 2021-09-13

**Authors:** Sol Gutiérrez Álvarez, Weihua Lin, Mohamed Abdellah, Jie Meng, Karel Žídek, Tõnu Pullerits, Kaibo Zheng

**Affiliations:** † Department of Chemistry, Technical University of Denmark, DK-2800 Kongens Lyngby, Denmark; ‡ Department of Chemical Physics and NanoLund Chemical Center, Lund University, P.O. Box 124, 22100 Lund, Sweden; § Department of Physical Chemistry, Uppsala University, Lägerhyddsvägen 1, 752 37 Uppsala, Sweden; ∥ The Research Centre for Special Optics and Optoelectronic Systems (TOPTEC), Institute of Plasma Physics, Czech Academy of Sciences v.v.i., Za Slovankou 1782/3, 182 00 Prague 8, Czech Republic

**Keywords:** ultrafast spectroscopy, diffusion lengths, CsPbBr_3_, DDAB, quantum dot photovoltaics, carrier transport, charge transfer

## Abstract

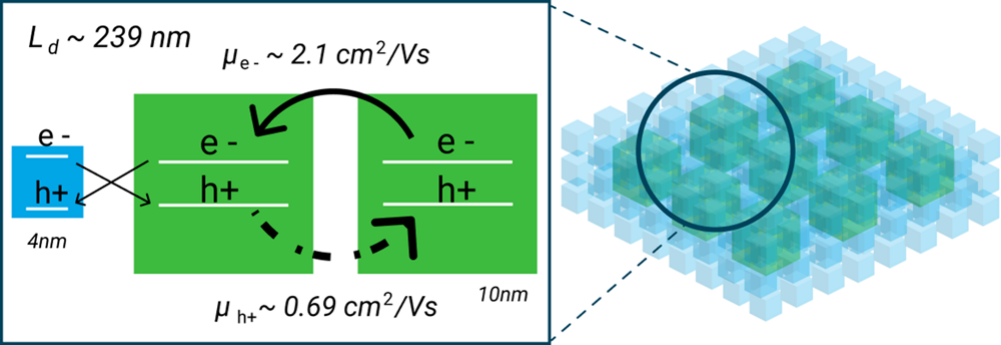

CsPbBr_3_ quantum dots (QDs) are promising candidates for optoelectronic devices. The substitution of oleic acid (OA) and oleylamine (OLA) capping agents with a quaternary alkylammonium such as di-dodecyl dimethyl ammonium bromide (DDAB) has shown an increase in external quantum efficiency (EQE) from 0.19% (OA/OLA) to 13.4% (DDAB) in LED devices. The device performance significantly depends on both the diffusion length and the mobility of photoexcited charge carriers in QD solids. Therefore, we investigated the charge carrier transport dynamics in DDAB-capped CsPbBr_3_ QD solids by constructing a bi-sized QD mixture film. Charge carrier diffusion can be monitored by quantitatively varying the ratio between two sizes of QDs, which varies the mean free path of the carriers in each QD cluster. Excited-state dynamics of the QD solids obtained from ultrafast transient absorption spectroscopy reveals that the photogenerated electrons and holes are difficult to diffuse among small-sized QDs (4 nm) due to the strong quantum confinement. On the other hand, both photoinduced electrons and holes in large-sized QDs (10 nm) would diffuse toward the interface with the small-sized QDs, followed by a recombination process. Combining the carrier diffusion study with a Monte Carlo simulation on the QD assembly in the mixture films, we can calculate the diffusion lengths of charge carriers to be ∼239 ± 16 nm in 10 nm CsPbBr_3_ QDs and the mobility values of electrons and holes to be 2.1 (± 0.1) and 0.69 (± 0.03) cm^2^/V s, respectively. Both parameters indicate an efficient charge carrier transport in DDAB-capped QD films, which rationalized the perfect performance of their LED device application.

## Introduction

CsPbBr_3_ colloidal perovskite quantum dots (QDs) have recently attracted great interest to the research community due to their outstanding optoelectronic properties.^1,2^ The facile solution-processed synthesis, size-tunable optical bandgap, and near-unity photoluminescence quantum yields (PLQYs), together with narrow emission bandwidth, render these materials great potential in different applications such as photodetectors, solar cells, and especially light-emitting diodes (LEDs).^1,3−6^

Despite substantial materials and device engineering, the transport of charge carriers in QD solids remains a challenge for LED applications.^6^ The conventional capping agents for CsPbBr_3_ QDs such as oleylamine (OLA) and oleic acid (OA) stabilize the QDs but prevent the carrier from hopping across the QDs in the solid. One promising solution is to replace them with smaller capping ligands, which has been done with molecules like octylamine^7^ or di-dodecyl dimethyl ammonium bromide (DDAB).^8,9^ DDAB is a quaternary alkylammonium with a halide ion pair that provides strong anchoring to the QD surface. This leads to superior surface passivation with an enhanced PLQY.^10,11^ In addition, shorter alkyl chains of DDAB compared with OA and OLA are believed to facilitate the charge carrier transport.^12,13^ Significant improvement in LED external quantum efficiency (EQE) using DDAB-capped QDs has been reported recently, with the recording value reaching 13.4%.^14,15^ However, the enhancement of the charge carrier transport by the DDAB capping agent has not been confirmed, with the important parameters including carrier mobility and diffusion coefficient.^4,16,17^ Furthermore, the assembly of the QDs in the solid plays a vital role in the charge transport in the solid, which should also be influenced by the surface capping agents. Therefore, a systematic characterization of the charge carrier transport dynamics in a DDAB-capping QD solid film is of great importance to rationalize the underlying mechanism of the optoelectronic devices.

There are several techniques to study charge carrier transport in QD solids. The electrical methods (e.g., field effect transistor and Hall effect measurements) are highly sensitive to electrode contact and device configuration.^18−20^ Instead, the optical methods focus more on the intrinsic features of the QD solid materials.^21−23^ Those methods usually introduce an acceptor to terminate the charge carrier diffusion and probe the lifetime of the charge carriers by ultrafast spectroscopies before they get trapped by the acceptors. In most cases, the target film is coated onto the acceptor layer, and different charge carrier lifetimes are obtained by varying the thickness of the target layer, which is defined as a 1D model.^24^ In the 3D model, donors and acceptors are intermixed and the diffusion path of the charge carrier is controlled by the inter-ratio between the donors and acceptors.^4,25^ Such a 3D model is closer to the functioning status in a real device.

In this paper, we use this 3D model to analyze the charge carrier transport dynamics in randomly mixed two-sized DDAB-capped CsPbBr_3_ QD solids (4 nm QD (QD-4) and 10 nm QD (QD-10)) by fs-TA. The ratio between two QDs varies to control the mean free path of the photogenerated charge carriers. Singular value decomposition (SVD) analysis first revealed that the carriers in QD-10 diffuse and get recombined with the carriers in QD-4. This means smaller QDs can be considered as acceptors in the bi-sized QD solid. We then vary the ratio between QD-10 and QD-4 and measure the mean diffusion time of electrons and holes in QD-10 clusters from the SVD fitting components of TA. In the next step, a Monte Carlo simulation was employed to model the assembly of the QDs and provided the average distance between the donor (QD-10) and the acceptor (QD-4). The diffusion coefficient and electron/hole mobility for QD-10 in the solids were then calculated using the 3D mixture diffusion model. The electron mobility obtained is (2.1 ± 0.1) cm^2^/Vs and the hole mobility is (0.69 ± 0.03) cm^2^/Vs. The diffusion length of the photoinduced charge carriers in the QD-10 obtained is 239 ± 16 nm. In contrast, a previous report shows that only energy transfer can occur in OA-capped QD solids with much lower *L*_D_ of exciton migration (50 nm).^26^ This confirms the improved charge carrier separation and transportation in DDAB-capped QD solids, which rationalize the enhanced device performance. Also, the Monte Carlo simulation shows how the concentration of the different sizes of QDs in the solid influences the average QD–QD distance and determines the interdot charge transfer efficiency. We believe that our results can be a useful guide of the film preparation as building blocks in the perovskite QD LED devices.

## Results and Discussion

Two sizes of DDAB-capped CsPbBr_3_ quantum dots (QDs) were employed in our study (i.e., 4 nm (QD-4) and 10 nm (QD-10)). They were synthesized by a previously reported hot injection method initially capped by OA and OLA and afterward underwent a surface ligand exchange to DDAB.^27^ The detailed synthesis procedure for QDs is provided in the Supporting Information.

**Figure 1:**
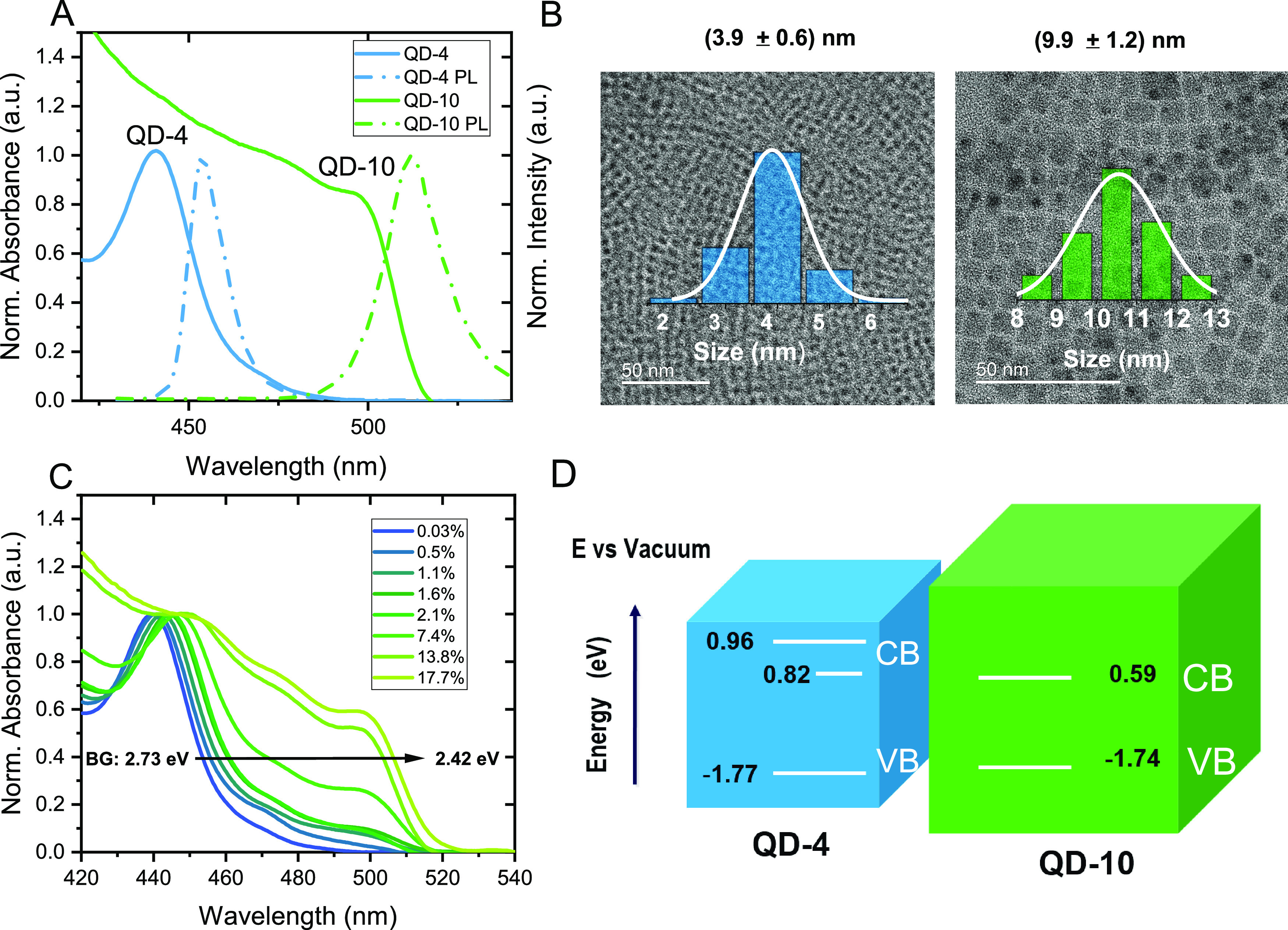
(A) Absorption (straight line) and photoluminescence (dotted line) spectra of pure QDs with sizes of 4 and 10 nm. (B) TEM images and size distribution of two sizes of QDs. (C) Absorption spectra of the mixture film with ascending percentage of 10 nm QD bandgap (BG) extracted from the Tauc plot in Figure S3. (D) Energy band alignment.

Figure 1A shows the UV–Vis absorption and photoluminescence spectra of each size of QDs in the colloidal solution form. The absorption band edges for QD-10 and QD-4 are located at 497 and 440 nm, respectively. The narrow emission bandwidth of both samples (i.e., 12.3 ± 0.3 nm for QD-4 and 21.6 ± 0.2 nm for QD-10) indicates their narrow size distribution. The mean sizes of 3.9 ± 0.6 and 9.9 ± 1.2 nm are further confirmed by HR-TEM measurements as seen in Figure 1B and Figure S1. To study the carrier diffusion dynamics, two quantum dots are used in the mixture film. It is commonly believed that larger QDs with a narrower bandgap can be considered as an acceptor to terminate the electron or hole transport due to the energetically favorable charge transfer from big QDs to small QDs.^24^ The photoexcited charge carriers in donor QDs (i.e., the smaller QDs) change their lifetime when the concentration of the acceptor varies. However, in our system, the situation is different where the big QDs serve as acceptors, which will be discussed later.

To fabricate the QD mixture films, we first mixed two QDs directly in the solution phase with the concentration ratio () varying from 0.3 to 5.1%. The solid films are then prepared by spin-coating 50 μL of the mixture onto quartz substrates. In the following text, all the multisized QD films are referred to by these *R*_QD_ percentage values.

The absorption spectra (Figure 1C) further confirm the mixture between QD-4 and QD-10 in those films with various ratios. The bandgap (BG) change is also depicted in Figure 1C and extracted from the Tauc plot in Figure S3, and the values of BG for each film are reported in Table S4. The redshift of the QD-4 exciton absorption band can also be observed in the film, with the increment of the QD-10 ratio attributed to the enhanced light scattering from the larger QDs. The energy band alignment between two QDs (Figure 1D) is confirmed by the XPS measurement to probe the valence band maximum (VBM) position (Figure S7) as well as Tauc plots of the absorption spectra (Figure S2) to determine the optical bandgap. The in-band state at 0.82 eV refers to a trap state located in QD-4, which will be explained later. The band alignment indicates that both electron transfer and hole transfer are energetically favorable from QD-4 to QD-10.

The photoinduced charge carrier dynamics in multisized QD films was investigated by transient absorption (TA) spectroscopy. A similar investigation has been implemented in PbS QD solids where one size of QDs is utilized as traps.^24^ The method is based on the concept that diffusing charge carriers are captured by those traps, determining the diffusion length and lifetime.^4^ Therefore, by introducing a fixed number of traps, the diffusion coefficient can be calculated by tracking the charge carrier lifetimes at various trap densities accordingly.

**Figure 2:**
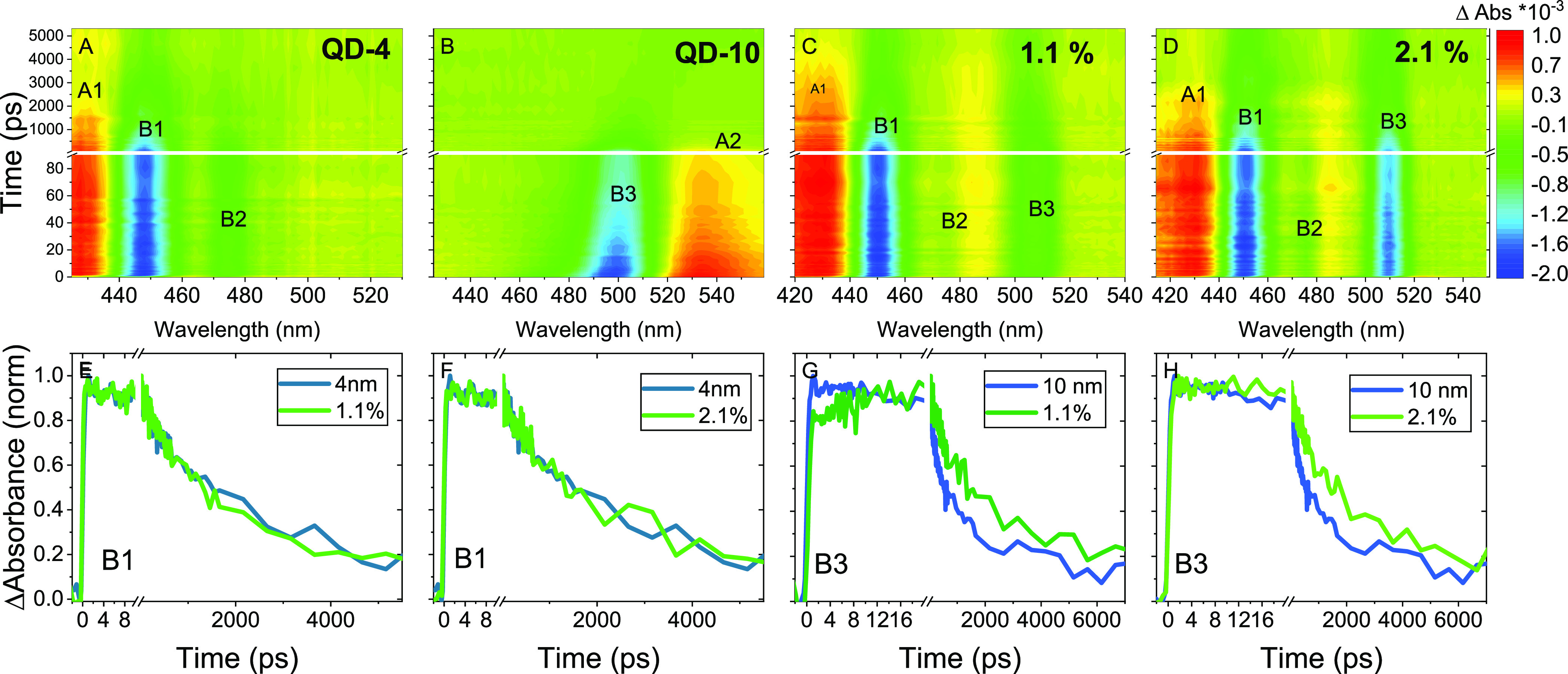
(A–D) TA spectrodiagram of QD-4, QD-10, and 1.1 and 2.1% mixture samples. Excitation pump at 400 nm. (E, F) TA kinetics of GSB B1 for QD-4 and 1.1 and 2.1% mixture samples. (G, H) TA kinetics of GSB for 10 nm QD and 1.1 and 2.1% mixture samples.

However, in the following, we will demonstrate that the charge carrier diffusion dynamics in multisized DDAB-capped CsPbBr_3_ QD solids is more complicated. Figure 2A,B shows the TA spectra of single-sized QD-4 and QD-10 films. Pronounced ground-state bleach (GB) at the band edge absorption regions B1 and B3 for QD- 4 and QD-10, respectively, can be attributed to the population of the lowest excited-state carriers. In addition, the TA spectrum of QD-4 presents an additional bleach at 470 nm (B2). It could be related to either the GBs of larger-sized QDs with a lower optical bandgap or a sub-bandgap trap state with optical strength for absorption. The TEM analysis of QD-4 can exclude the existence of another distinct size of QDs. Therefore, the B2 band should be attributed to the trap state filling. This can be further verified by the observable absorption tail after the excitonic absorption band edge from 460 to 480 nm in Figure 1A. Such absorptive sub-bandgap trap states have been widely reported in lead halide bulk materials.^28,29^

In the mixture films, both QD-4 and QD-10 are excited by a 400 nm excitation laser pulse. Therefore, the GB of the band edge exciton transition of the two QDs, i.e. B1, B2, and B3, occurs concurrently as shown in Figure 2C,D. Figure 2C,D demonstrates the TA spectra of mixture films with two typical *R*_QD_ values of 1.1 and 2.1%.

To reveal the charge transport dynamics, we first compared the TA kinetic traces at B1 and B3 in both mixed films with the neat QD-4 and QD-10 films (Figure 2E–H). The kinetic traces at B1 exhibit a negligible difference between the mixed and neat QD films (Figure 2E,F). On the contrary, TA kinetic traces at B3 corresponding to the excited-state population of QD-10 exhibit a difference between the mixed films and the neat QD-10 film as shown in Figure 2G,H. At the early timescale, the B3 kinetics in the 1.1% film shows a slow rising component up to 14 ps compared with the instantaneous rise of the signal in the pure QD-10 film in Figure 2G. Such a slow rise is less pronounced in the 2.1% film as well as in other mixed films as shown in Figure 2H and Figure S4B. In general, the slower buildup of the TA GB in the mixture system compared with the single components indicates the charge injection from the donor to the acceptor. Unique early time rising kinetics in the 1.1% film suggests different charge transfer dynamics from the other mixed film, which will be discussed later. At the longer timescale, the TA kinetics of B3 decays slower in mixed films than the neat QD-10 film, with the decay rate increasing with the decrease in QD-10 concentration. This can be attributed to the delayed charge transfer after the charge carrier diffusion in the donor observed in the multisized QD film system.^24^

**Figure 3:**
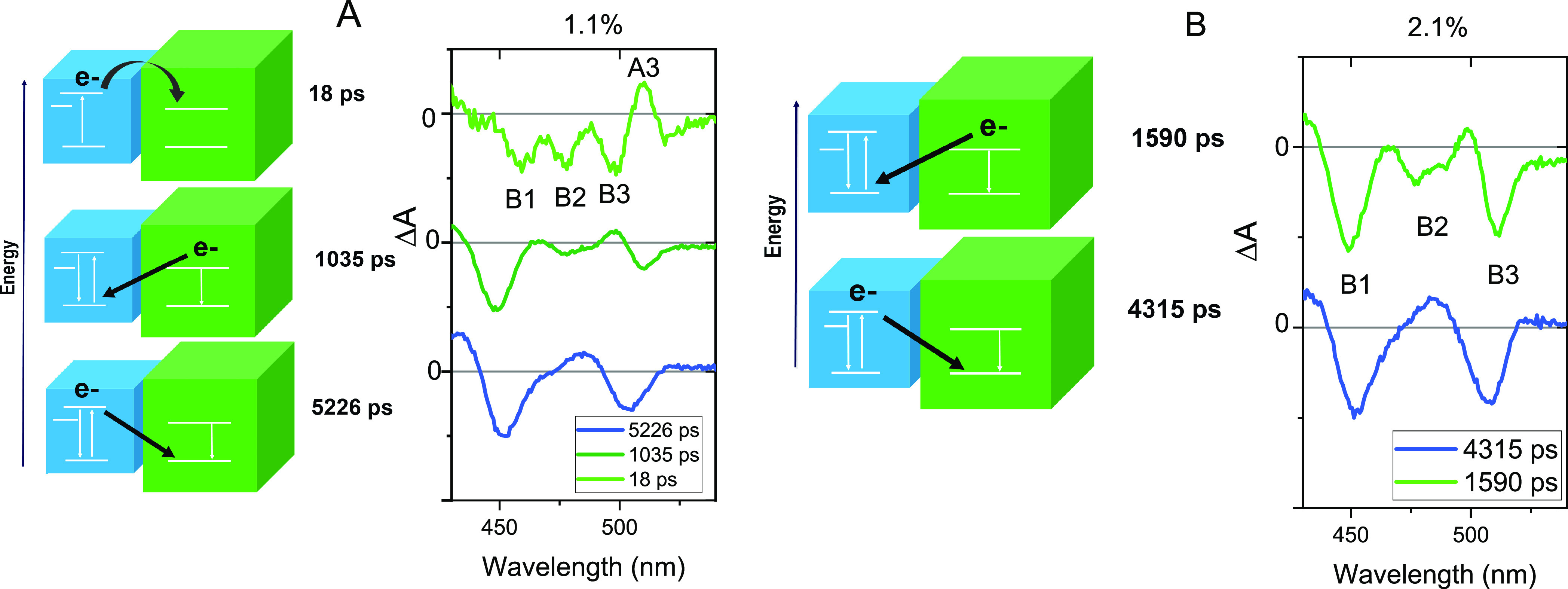
SVD components of mixture films and the diagram of the process of decay-associated spectra (DAS) at (A) 1.1% and (B) 2.1%.

The above single-wavelength kinetics manifests the diffusion-assisted interfacial charge transfer in our multisized QD films. To unambitiously assign each photoinduced process in the mixture film, we carried out the singular value decomposition (SVD) of the TA dynamics where the decay-associated spectra (DAS) have been extracted. Figure 3 summarizes the different behaviors for the 1.1% mixture and 2.1% mixture from the SVD analysis.

In the 1.1% film, we can extract three DAS components: one short-lived component with a lifetime of 18 ps and two long-lived components with lifetimes of 950 and 5022 ps. The lifetime of the fastest component (18 ps) resembles the slow rising time of B3 kinetics in the mixture film, as shown in Figure 2G. The negative bands at 450 and 480 nm represent the decay of B1 and B2. The positive band at 508 nm together with the negative band at 495 nm mirrors the excited-state absorption (A3), representing the rise of A3 and GB of QD-10 (B3), respectively. Therefore, they refer to the appearance of A3 and B3 in QD-10, which can be further confirmed by the TA kinetics at these wavelengths (more details in Figure S5). Such simultaneous excited-state depopulation in QD-4 with the population in QD-10 is a fingerprint of interdot electron injection or energy transfer^30^ from QD-4 to QD-10. However, since the exciton binding energy of CsPbBr_3_ QDs is in the order of thermal energy at room temperature, the majority of the photoexcited species should be free carriers in the solids.^16^ In addition, electron transfer should be more reasonable at the rates that are observed.^31^

The second component with a lifetime of 1035 ps shows the negative bands at B1, B2, and B3 corresponding to the decay of three GBs at the same time. The excited-state depopulation dynamics within such a lifetime is absent in both individual QD-4 and QD-10 (for details, see Figure S6). Therefore, such simultaneous depopulation of the excited state in two QDs can only be attributed to the interdot charge recombination. Since this recombination also induces the decay of the trap state bleach B2, it must be the recombination of the excited electrons in QD-10 with the excited holes in QD-4. This is because the hole population at VB in QD-4 would also block absorption transition to the trap states, whereas the electron population at CB is independent of such a process. On the other hand, the component with the longest lifetime of 5 ns featured only a negative band of B1 and B3. Therefore, it should be attributed to the recombination of the excited electrons in QD-4 and the excited holes in QD-10 instead. The above recombination process takes into account the time for photogenerated carriers in one size of QD to diffuse to the interface with the other size of QDs. Therefore, the DAS component’s lifetime can be utilized to analyze the charge carrier diffusion with various *R*_QD_ values. The 2.1% mixed film, for instance, exhibits the same two interfacial charge recombination components in SVD fitting but with different lifetimes (Figure 3B). This phenomenon applies to the rest of the mixed films, indicating an *R*_QD_-dependent charge carrier diffusion in the mixture film given that the interfacial charge recombination time is independent of the QD assembly. However, we did not observe the ultrafast electron injection component from small QDs to large QDs in the other mixture film. The detailed reason needs to be further explored. The trap state denoted by B2 can either be an electron trap or a hole trap. However, if B2 refers to the hole trapping, we should obtain a 1000 ps hole diffusion time and 5000 ps electron diffusion time from the SVD signal. Despite the similar effective mass of the electron and hole in CsPbBr_3_ perovskites, the mobility of the electron in such a perovskite should still be expected to be larger than the hole due to polaron formation.^32^ According to a previous study, excitation in CsPbBr_3_ would generate large electron polarons and small hole polarons, which should lead to a larger electron mobility than the hole.^32^ In this scenario, we believe that the overall electron diffusion should be more efficient than the hole diffusion, and thus, the above situation to assign B2 as hole traps can be excluded.^10^

**Figure 4:**
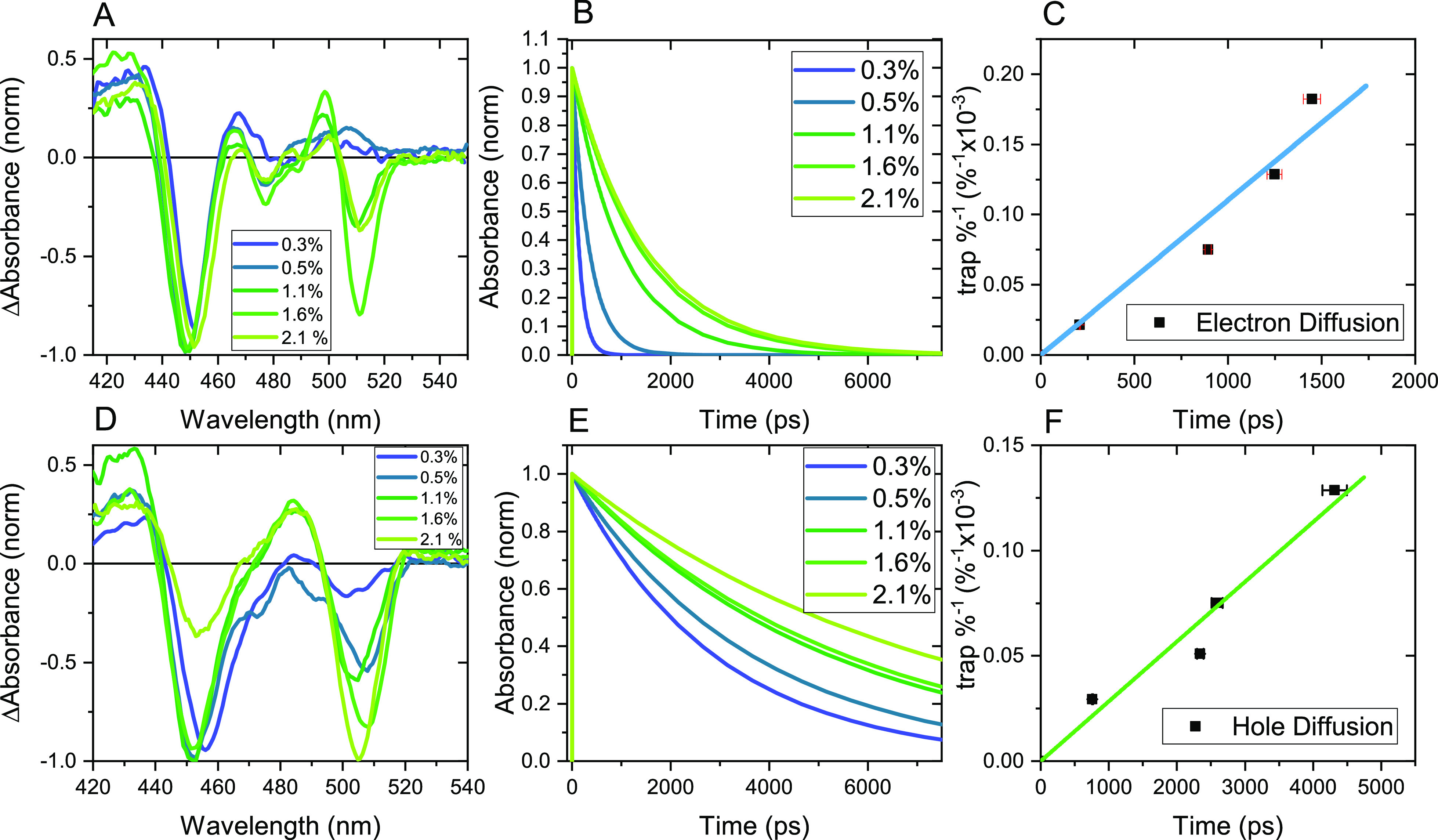
(A) DAS of the electron recombination process for ascending *R*_QD_; (B) SVD-associated kinetic trace for the DAS of the electron recombination process with ascending *R*_QD_; (C) % trap vs trapping time plot describing the electron diffusion process; (D) DAS of the hole recombination process for ascending *R*_QD_; (E) SVD-associated kinetic trace for the DAS of the hole recombination process with ascending *R*_QD_; (F) % trap vs trapping time plot describing the hole diffusion process.

In the next step, we aim to reveal the dependence of QD-10 concentration on charge diffusion dynamics. We observed that the lifetime of the two long-lived components is increasing with the increment of QD-10 concentration in the mixture film but keeping the primary spectral feature shown in Figure 4A,D with the corresponding SVD fitted component decay trace plotted in Figure 4B,E. This indicates the prolongation of the charge carrier diffusion as discussed above. Figure 4C,F shows the *N*_t_^–1^ vs τ_trap_, which will be used for the diffusion parameter calculation later. Interestingly, we notice the same trend in the single-wavelength TA kinetics at B3, but the TA kinetics at B1 almost remains constant as shown in Figure S4A. It occurs together with the reduction of the relative amplitude ratio between B1 and B3 bands in the DAS component, as shown in Figure 4A. All the above phenomena denote that changes in TA dynamics should be dominated by the photophysical processes in QD-10 rather than in QD-4. According to the literature reports, the exciton binding energy (*E*_b_) of large-sized CsPbBr_3_ QDs (i.e., 8 nm) is about 40 meV,^3,33^ meaning that the major excited species should be dissociated free carriers according to the Saha–Langmuir model.^34^ On the other hand, a larger *E*_b_ as well as numerous surface trap states in smaller QDs is expected to largely hinder the carrier diffusion.^35^

**Figure 5:**
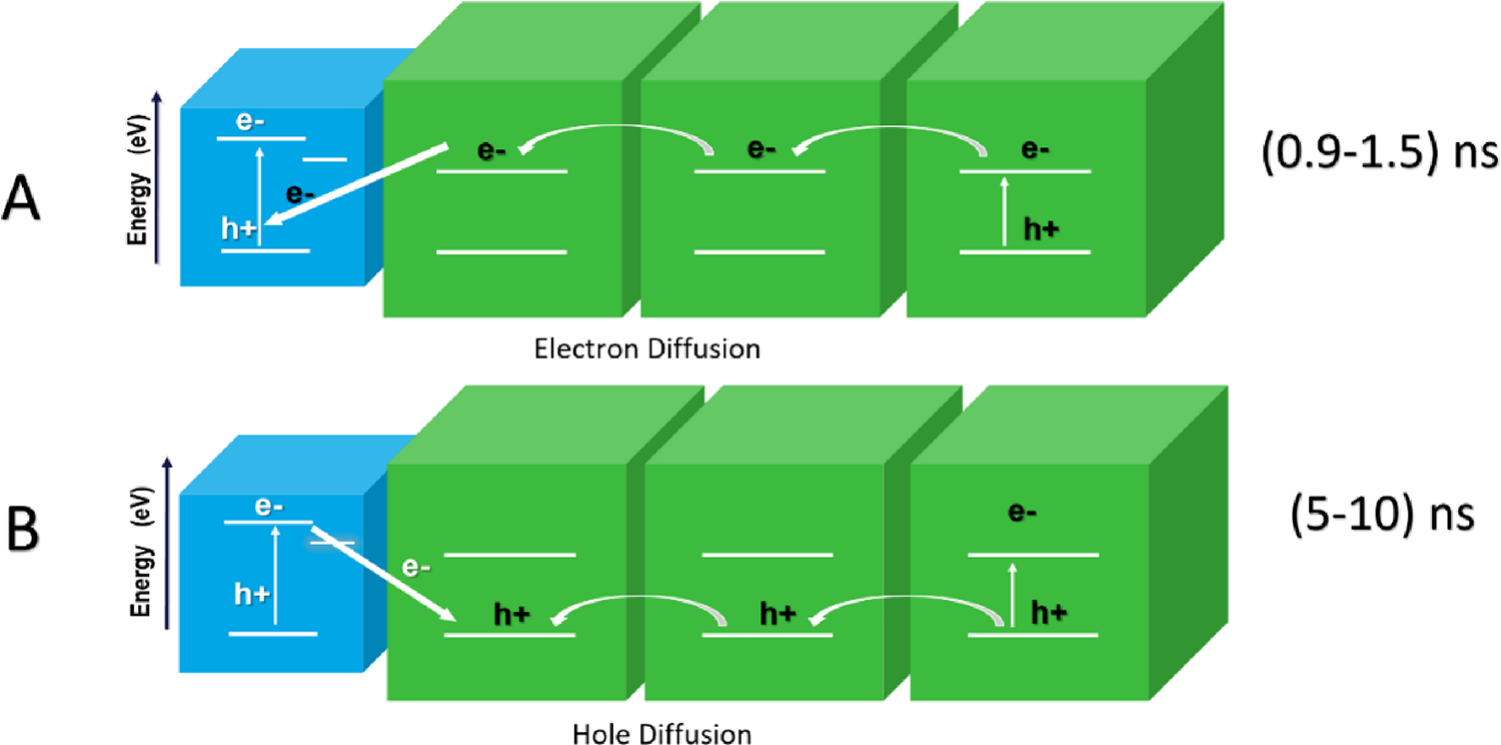
(A) Schematic of the diffusion in 4 and 10 nm QD mixtures in our system. (B) Schematic of the excited-state dynamics between 4 and 10 nm QDs.

In addition, the charge recombination at the QD-4 and QD-10 interface is mainly decided by the interdot spacing as well as the energetic driving force extracted from the energy band alignment between two QDs. All those factors are independent of the QD-10 ratio. Consequently, the variation on the overall charge recombination time extracted from the TA dynamics can only be induced by the different charge carrier diffusion time prior to the interfacial recombination, as illustrated in Figure 5A,B.

**Figure 6:**
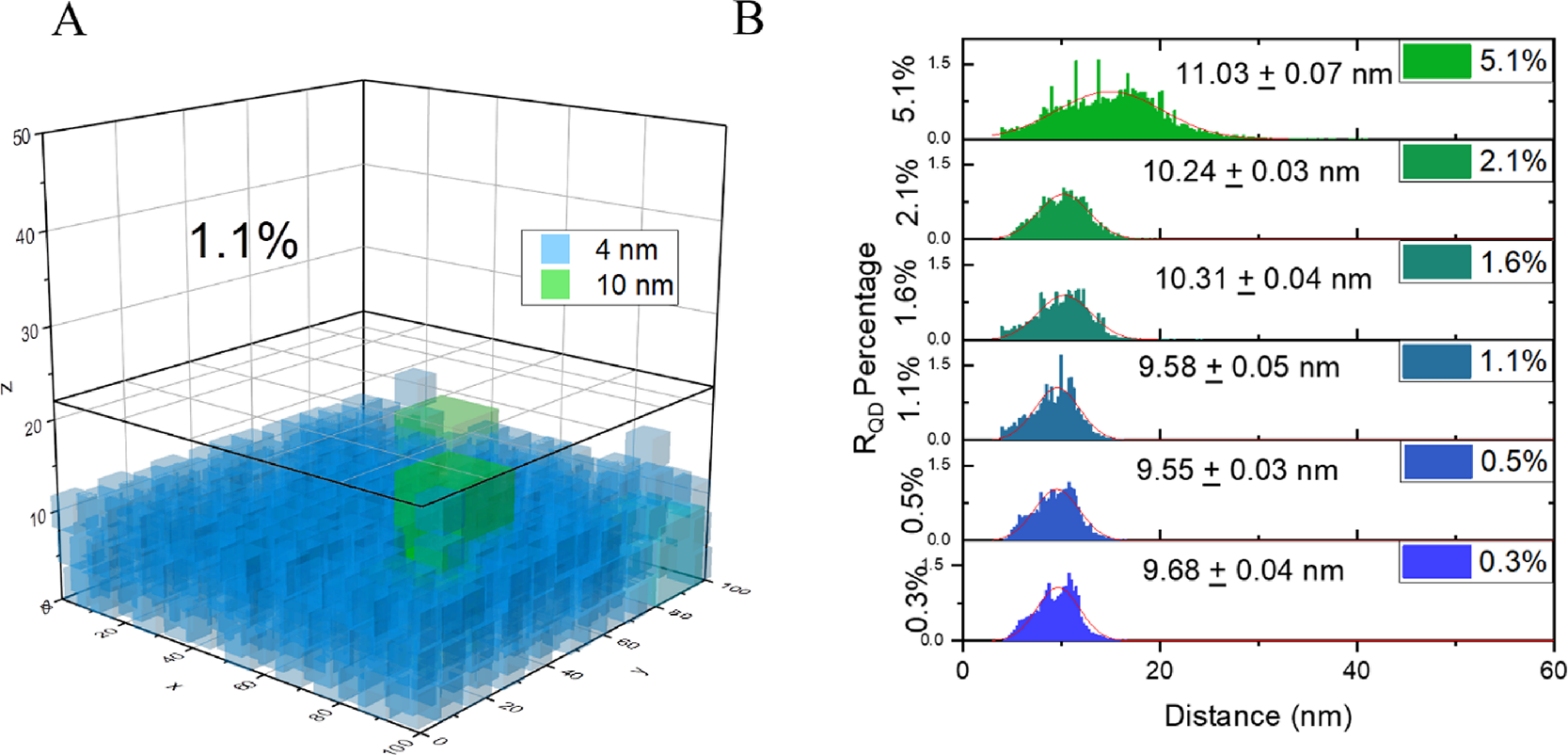
(A) Monte Carlo simulation for two 1.1% *R*_QD_ layers of 100 nm^2^ set for a minimum deposit of 1000 QDs and a maximum of 5000 QDs. (B) Histogram of the average distance of the first 20 nearest QD-10 to QD-4.

When more QD-10 are integrated into the mixture film, the average distance between QD-4 and QD-10 would increase as the aggregated domain of the QD-10 cluster starts to expand. This should prolong the diffusion path length for the charge carrier in QD-10 to the interface of QD-4. We can evaluate the average QD center-to-center distance between QD-4 and QD-10 in the random mixture film using a Monte Carlo simulation on the internal assembly of the QD.^5,7^ The initial input for the simulation includes the total amount of quantum dots, the geometry, and the size of both quantum dots QD-4 (4 nm) and QD-10 (10 nm) (Figure 6A) (for details of the simulation, see the Supporting Information). The histograms of the first 20 nearest neighbors of QD-10 to QD-4 were summed to provide the overall statistics for different *R*_QD_ values (Figure 6B). The simulated distribution of the *R*_QD_ represents the packaging capacity of the QD assembly. As observed in Figure 6B, the average D–A distance varies little from 0.3 to 1.1% sample, followed by a pronounced distance increasing with the increment in the Q-10 concentration above 1.1%. Such a trend can also be observed in the full width half maximum (FWHM) as summarized in Table S3, where the QD center-to-center distance distribution broadens with the increase of QD-10 (Figure 6B).

After knowing the average diffusion path length in the mixture film, we can determine the diffusion coefficient of electrons and holes by applying a modified version of the Zhitomirsky 3D model for carrier diffusion.^4^ This model modulates the carrier diffusion by controlling the population of acceptors (i.e., traps) in the mixture. However, unlike in the conventional case where the narrow bandgap QDs can be considered as charge carrier acceptors, in our CsPbBr_3_ QD mixture film, QD-4 serves as an acceptor for larger-sized QD-10 since the interfacial Z-scheme charge recombination is more dominant than charge injection within the CB or VB.

To implement the Zhitomirsky 3D modeling, we need to establish the relationship between the lifetimes of diffused charge carriers in the QD solid extracted from the SVD component and the calculated trap percentage. As discussed above, we simplify the hole and electron recombination lifetimes obtained in two SVD components to be the diffusion time of two species to the acceptor’s interface (i.e., QD-4).

The capture rate of carriers into traps (*k*_trap_) is the inverse of the trapping lifetime τ_trap_^–1^ and, in the Shockley–Read–Hall recombination model, can be expressed as^24^
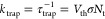
1where *V*_th_ is the thermal velocity in the hopping regime, also expressed as *V*_th_ = *d*/τ_hop_ with the interdot distance *d* and the interdot hopping time τ_hop_. *N*_t_ is the density of traps. σ is the capture cross section, which for the 3D model is assumed to be .^4^ Mobility μ is expressed as
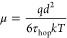
2where *k* is the Boltzmann distribution, *T* is the temperature, and *q* is the charge of the carrier.

*D* is related to carrier mobility via the Einstein relation
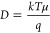
3

From the Einstein equation relating diffusion *D* and μ
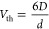
4

*D* can be obtained as
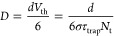
5

By rearranging this formula, we obtain
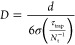
6

Therefore, we can obtain *D* from the slope in the plot of *N*_t_^–1^ vs τ_trap_ (Figure 4C,F). Since the diffusion process in both cases occurs in QD-10, *N*_t_^–1^ refers to the percentage of QD-4. The interdot distance (12 nm) and QD density (3.8 × 10^17^ cm^–3^) of the pure QD-10 film are obtained from the Monte Carlo simulation (for details of these parameters, see the Supporting Information). Table 1 summarizes the calculated diffusion parameters.

**Table 1:** Transport Properties for the QDs Obtained from the Diffusion Model and Comparison of Diffusion Measurements for the CsPbBr_3_ QD

Method	Sample	State	Capping agent	Acceptor	μ	L_d_	(cm^2^/s)	Slope (% trap^–1^ ps^–1^)
TPLQ*^26^	halide-treated CsPbBr_3_ QDs	solid film	OA	Au Np	I^–^: 0.009 Cl^–^: 0.018 cm^2^/s	I^–^: 52 and Cl^–^: 71 nm		
T-THz^**^^16^	11 nm CsPbBr_3_ QDs	sls (HMN)^a^	OA	N.A.	4500 cm^2^/V s	(>9.2 μm)		
TAS (this study)	10 nm CsPbBr_3_ QDs	solid film	DDAB	4 nm CsPbBr_3_	e^–^: (2.1 ± 0.1) h^+^: (0.69 ± 0.03) cm^2^/V s	239 ± 15 nm	e^–^: (5.3 ± 0.4) × 10^–2^; h^+^: (1.76 ± 0.07) × 10^–2^	e^–^: ( 1.16 ± 0.08) × 10^–5^; h^+^: (3.8 ± 0.1) × 10^–6^

a 2,2,4,4,6,8,8-Heptamethylnonane. *Time resolved fluorescence quenching. **Time resolved THz.

The diffusion length (*L*_d_) was calculated from the diffusion coefficient *D* of the carriers and their lifetime by the following formula: . The diffusion length relies on the density of acceptors in our QD system. Using the carrier lifetime of the neat QD-10 film (10.7 ns), we can obtain an *L*_d_ of 239 ± 16 nm. The diffusion coefficient and corresponding mobility of the electrons are higher than those of the holes, which is consistent with the argumentation above.

As summarized in Table 1, the carrier diffusion length for the DDAB-capped CsPbBr_3_ samples in our study is significantly longer than the values in the OA-capped CsPbBr_3_ solid samples. On the other hand, we notice that the diffusion length obtained by the THz method shows a very high value in comparison with our result. This is because the THz measurement only reflects the intrinsic capabilities of the charge transport dominated by the local acoustic phonon or optical phonon scattering and is less capable of tracking the scattering with defects or interfacial boundaries, which can dominate in the QD solid film.^36^

## Conclusions

We studied the electron and hole transport dynamics in densely packed QD films using two different sizes of quaternary alkylammonium-capped CsPbBr_3_ QD mixtures via the TA spectroscopy analysis. The excited-state dynamics in the TA measurement exhibits strong dependence on the QD ratio. Using the SVD analysis, we reveal that the photoinduced electrons and holes are less mobile in small-sized QD-4, whereas they are more freely diffused within QD-10 clusters and recombine with charges in QD-4 at the interface. The lifetimes of such diffused charge carriers are decided by the ratio of QD-10 (i.e., the mean path length between QD-10 and QD-4). After simulating such a mean path length using a Monte Carlo simulation with different QD-10 ratios in the mixture film, we conduct Zhitomirsky 3D modeling for carrier diffusion, which provided the charge carrier diffusion coefficients of electrons and holes in QD-10 of (5.3 ± 0.4) × 10^–2^ and (1.76 ± 0.07) × 10^–2^ cm^2^/s, respectively. The calculated charge carrier diffusion length is longer than the reported value for OA/OLA-capped QD films by a factor of 5, suggesting enhanced charge carrier transport dynamics. We argue that perovskite QD solid-based optoelectronic devices can highly benefit from such an understanding of the relationship between charge transport dynamics and film configuration as well as QD surface ligands. The characterization methodology in this paper can be also applied to optoelectronic devices constructed by the mixture of different nanocrystals or even plasmonic nanoparticles in the photoactive layers where the mean free path of the excited charge carriers plays a critical role for the device performance.

## Experimental Section

### Synthesis Method

CsPbBr_3_ QDs of two different band gaps and sizes were synthesized following a previously published procedure.^1^ The ligand exchange follows a method described elsewhere.^2^

### Materials

CsCO_3_ (99.9%), octadecene (ODE; for synthesis), oleic acid (OA; technical grade, 70%), PbBr_2_ (99.999%), oleylamine (OLA; technical grade, 70%), toluene (99.8%), hexane (95%), and di-dodecyl dimethyl ammonium bromide (DDAB; 98%) were purchased from Sigma-Aldrich. ODE, OA, and OLA were degassed at 120 °C before any reaction.

### Cesium Oleate Synthesis

In a 50 mL round-bottom flask, 0.407 g of CsCO_3_ was added to 20 mL of ODE and 1.25 mL of OA. This mixture was degassed for 1 h at 120 °C and then set under argon for 30 min at 150 °C. This was preheated to 100 °C for the quantum dot synthesis.

### Synthesis of Quantum Dots

In a 50 mL three-necked round-bottom flask, 0.1378 g of PbBr_2_ was added to 20 mL of ODE and degassed under vacuum for 1 h. The mixture was then set under inert conditions (Ar) at 120 °C; afterward, 1 mL of OLA and 1 mL of OA were injected, and the temperature was changed to 150 °C until PbBr_2_ was completely soluble. The injection temperature of the mixture was then increased for different sizes (140 °C for 4 nm and 180 °C for 10 nm). When the temperature was reached, 0.8 mL of cesium oleate was added swiftly, and the mixture was immediately put on an ice bath.

### Purification for QD-4

The reaction mixture was centrifuged for 10 min at 6500 rpm, and the supernatant was collected and precipitated within a 3:1 methyl acetate/supernatant mixture. This was centrifuged for 10 min at 6500 rpm, and the precipitate was collected and dissolved in 10 mL of toluene. This is the QD-4 solution in OA.

### Purification for QD-10

The reaction mixture was centrifuged for 10 min at 6500 rpm, and the precipitate was collected and redissolved in 10 mL of toluene. The mixture was then centrifuged for 15 min at 5000 rpm, and the supernatant was than collected. This is the QD-10 solution in OA.

### Ligand Exchange

This procedure was based on a reported method elsewhere. We added 500 μL of OA and 1000 μL of 0.05 M solution of DDAB in hexane. This was shaken and then precipitated with methyl acetate. The precipitate was redissolved in 5 mL of hexane. This is the stock solution.

### Film Preparation

Quartz slides (1 × 1 cm) were cleaned with sequential sonication in acetone and isopropanol each for 20 min and then ozone-cleaned with plasma. QD films were deposited by spin-coating 50 μL of the QD mixtures with *R*_QD_ values ranging from 0.32 to 7.44% at 1000 rpm onto the cleaned quartz substrates.

### Absorption Spectroscopy

UV–Vis absorption spectra for colloidal solutions and films were collected using a spectrophotometer from Agilent Technologies (Santa Clara, USA).

### Photoluminescence Spectroscopy

The emission spectra steady-state photoluminescence was measured using a FluoroMax@-4 spectrofluorometer (HORIBA JOBIN YVON, Inc., Edison, NJ) with the excitation at 400 nm.

### Transmission Electron Microscopy

Imaging was conducted on a Tecnai G2 T20 TEM and FEI Titan Analytical 80-300ST TEM from FEI Company.

The obtained TEM images were processed with Gatan Microscopy Suite software.

### X-ray Photoelectron Spectroscopy

X-ray photoelectron spectroscopy (XPS; Thermo Scientific) was performed to analyze the valence and composition of the samples, with Al Kα (1486 eV) as the excitation X-ray source. The peak of C 1s at about 284.8 eV was used to calibrate the energy scale. The pressure of the analysis chamber was maintained at 2 × 10^–10^ mbar during measurement.

### Transient Absorption

TA experiments were performed by using a femtosecond pump-probe setup. Laser pulses (800 nm, 150 fs pulse length, and 3 kHz repetition rate) were generated by a Ti:sapphire amplifier with an integrated oscillator and pump lasers (Libra LHE, Coherent Inc.) and a transient absorption spectrometer (Newport Corp.). Briefly, the output of a Ti:sapphire amplifier with an integrated oscillator and pump lasers (800 nm, 150 fs, 3 kHz, Libra LHE, Coherent Inc.) was split into two beams that were used to generate 400 nm light through the doubling crystal as a pump beam and to generate white light through a CaF_2_ crystal as a probe. The probe beam was split to two beams: one going through the sample and another as a reference. The generated supercontinuum was then focused onto the sample and overlapped with the pump beam. The transient spectra were detected with a fiber-coupled CCD-based monochromator (Oriel, Newport). Samples for transient absorption experiments were kept in the dark between each measurement. Global SVD analysis was performed with the Glotaran software package (http://glotaran.org).

## Supporting Information Available

Calculations of the QD concentration, simulation details, TEM images, Tauc plot, kinetic traces at B1 and B3 at different *R*_QD_ values, decay-associated spectra and kinetic traces of pure QD films with sizes of 4 and 10 nm, and XPS spectra (PDF)

## References

[ref1] Snaith H. J. Present Status and Future Prospects of Perovskite Photovoltaics. Nat. Mater. 2018, 17, 372–376. 10.1038/s41563-018-0071-z. 29686248

[ref2] Stranks S. D.; Snaith H. J. Metal-Halide Perovskites for Photovoltaic and Light-Emitting Devices. Nat. Nanotechnol. 2015, 10, 391–402. 10.1038/nnano.2015.90. 25947963

[ref3] Protesescu L.; Yakunin S.; Bodnarchuk M. I.; Krieg F.; Caputo R.; Hendon C. H.; Yang R. X.; Walsh A.; Kovalenko M. V. Nanocrystals of Cesium Lead Halide Perovskites (CsPbX_3_, X = Cl, Br, and I): Novel Optoelectronic Materials Showing Bright Emission with Wide Color Gamut. Nano Lett. 2015, 15, 3692–3696. 10.1021/nl5048779. 25633588 PMC4462997

[ref4] Zhitomirsky D.; Voznyy O.; Hoogland S.; Sargent E. H. Measuring Charge Carrier Diffusion in Coupled Colloidal Quantum Dot Solids. ACS Nano 2013, 7, 5282–5290. 10.1021/nn402197a. 23701285

[ref5] Hu Z.; Liu Z.; Zhan Z.; Shi T.; Du J.; Tang X.; Leng Y. Advances in Metal Halide Perovskite Lasers : Synthetic Strategies , Morphology Control , and Lasing Emission. Adv. Photonics 2021, 3, 1–23. 10.1117/1.AP.3.3.034002.

[ref6] Cai W.; Chen Z.; Chen D.; Su S.; Xu Q.; Yip H. L.; Cao Y. High-Performance and Stable CsPbBr_3_ Light-Emitting Diodes Based on Polymer Additive Treatment. RSC Adv. 2019, 9, 27684–27691. 10.1039/C9RA05270D. PMC9070759 35529194

[ref7] Xiong Q.; Huang S.; Du J.; Tang X.; Zeng F.; Liu Z.; Zhang Z.; Shi T.; Yang J.; Wu D.; Lin H. Surface Ligand Engineering for CsPbBr_3_ Quantum Dots Aiming at Aggregation Suppression and Amplified Spontaneous Emission Improvement. Adv. Opt. Mater. 2020, 8, 1–8. 10.1002/adom.202000977.

[ref8] Chiba T.; Kido J. Lead Halide Perovskite Quantum Dots for Light-Emitting Devices. J. Mater. Chem. C 2018, 6, 11868–11877. 10.1039/C8TC03561J.

[ref9] Chiba T.; Hoshi K.; Pu Y. J.; Takeda Y.; Hayashi Y.; Ohisa S.; Kawata S.; Kido J. High-Efficiency Perovskite Quantum-Dot Light-Emitting Devices by Effective Washing Process and Interfacial Energy Level Alignment. ACS Appl. Mater. Interfaces 2017, 9, 18054–18060. 10.1021/acsami.7b03382. 28485139

[ref10] Zheng W.; Wan Q.; Liu M.; Zhang Q.; Zhang C.; Yan R.; Feng X.; Kong L.; Li L. CsPbBr_3_ Nanocrystal Light-Emitting Diodes with Efficiency up to 13.4% Achieved by Careful Surface Engineering and Device Engineering. J. Phys. Chem. C 2021, 125, 3110–3118. 10.1021/acs.jpcc.0c11085.

[ref11] Pan J.; Quan L. N.; Zhao Y.; Peng W.; Murali B.; Sarmah S. P.; Yuan M.; Sinatra L.; Alyami N. M.; Liu J.; Yassitepe E.; Yang Z.; Voznyy O.; Comin R.; Hedhili M. N.; Mohammed O. F.; Lu Z. H.; Kim D. H.; Sargent E. H.; Bakr O. M. Highly Efficient Perovskite-Quantum-Dot Light-Emitting Diodes by Surface Engineering. Adv. Mater. 2016, 28, 8718–8725. 10.1002/adma.201600784. 27529532

[ref12] Shamsi J.; Urban A. S.; Imran M.; De Trizio L.; Manna L. Metal Halide Perovskite Nanocrystals: Synthesis, Post-Synthesis Modifications, and Their Optical Properties. Chem. Rev. 2019, 119, 3296–3348. 10.1021/acs.chemrev.8b00644. 30758194 PMC6418875

[ref13] Van Le Q.; Jang H. W.; Kim S. Y. Recent Advances toward High-Efficiency Halide Perovskite Light-Emitting Diodes: Review and Perspective. Small Methods 2018, 2, 1700419 10.1002/smtd.201700419.

[ref14] Song J.; Li J.; Li X.; Xu L.; Dong Y.; Zeng H. Quantum Dot Light-Emitting Diodes Based on Inorganic Perovskite Cesium Lead Halides (CsPbX_3_). Adv. Mater. 2015, 27, 7162–7167. 10.1002/adma.201502567. 26444873

[ref15] Shynkarenko Y.; Bodnarchuk M. I.; Bernasconi C.; Berezovska Y.; Verteletskyi V.; Ochsenbein S. T.; Kovalenko M. V. Direct Synthesis of Quaternary Alkylammonium-Capped Perovskite Nanocrystals for Efficient Blue and Green Light-Emitting Diodes. ACS Energy Lett. 2019, 4, 2703–2711. 10.1021/acsenergylett.9b01915. 31737780 PMC6849336

[ref16] Yettapu G. R.; Talukdar D.; Sarkar S.; Swarnkar A.; Nag A.; Ghosh P.; Mandal P. Terahertz Conductivity within Colloidal CsPbBr_3_ Perovskite Nanocrystals: Remarkably High Carrier Mobilities and Large Diffusion Lengths. Nano Lett. 2016, 16, 4838–4848. 10.1021/acs.nanolett.6b01168. 27367476

[ref17] De Weerd C.; Gomez L.; Zhang H.; Buma W. J.; Nedelcu G.; Kovalenko M. V.; Gregorkiewicz T. Energy Transfer between Inorganic Perovskite Nanocrystals. J. Phys. Chem. C 2016, 120, 13310–13315. 10.1021/acs.jpcc.6b04768.

[ref18] Dong Q.; Fang Y.; Shao Y.; Mulligan P.; Qiu J.; Cao L.; Huang J. Electron-Hole Diffusion Lengths > 175 m m in Solution-Grown CH_3_NH_3_PbI_3_ Single Crystals. Science 2015, 347, 967–970. 10.1126/science.aaa5760. 25636799

[ref19] Shi D.; Adinolfi V.; Comin R.; Yuan M.; Alarousu E.; Buin A.; Chen Y.; Hoogland S.; Rothenberger A.; Katsiev K.; Losovyj Y.; Zhang X.; Dowben P. A.; Mohammed O. F.; Sargent E. H.; Bakr O. M. Low Trap-State Density and Long Carrier Diffusion in Organolead Trihalide Perovskite Single Crystals. Science 2015, 347, 519–522. 10.1126/science.aaa2725. 25635092

[ref20] Herz L. M. Charge-Carrier Mobilities in Metal Halide Perovskites: Fundamental Mechanisms and Limits. ACS Energy Lett. 2017, 2, 1539–1548. 10.1021/acsenergylett.7b00276.

[ref21] Xing G.; Mathews N.; Lim S. S.; Lam Y. M.; Mhaisalkar S.; Sum T. C. Long-Range Balanced Electron- and Hole-Transport Lengths in Organic-Inorganic CH_3_NH_3_PbI_3_. Science 2013, 342, 344–347. 10.1126/science.1243167. 24136965

[ref22] Stranks S. D.; Eperon G. E.; Grancini G.; Menelaou C.; Alcocer M. J. P.; Leijtens T.; Herz L. M.; Petrozza A.; Snaith H. J. Electron-Hole Diffusion Lengths Exceeding 1 Micrometer in an Organometal Trihalide Perovskite Absorber. Science 2013, 342, 341–344. 10.1126/science.1243982. 24136964

[ref23] Eperon G. E.; Stranks S. D.; Menelaou C.; Johnston M. B.; Herz L. M.; Snaith H. J. Formamidinium Lead Trihalide : A Broadly Tunable Perovskite for Efficient Planar Heterojunction Solar. Energy Environ. Sci. 2014, 7, 982–988. 10.1039/c3ee43822h.

[ref24] Proppe A. H.; Xu J.; Sabatini R. P.; Fan J. Z.; Sun B.; Hoogland S.; Kelley S. O.; Voznyy O.; Sargent E. H. Picosecond Charge Transfer and Long Carrier Diffusion Lengths in Colloidal Quantum Dot Solids. Nano Lett. 2018, 18, 7052–7059. 10.1021/acs.nanolett.8b03020. 30359524

[ref25] Zhitomirsky D.; Voznyy O.; Levina L.; Hoogland S.; Kemp K. W.; Ip A. H.; Thon S. M.; Sargent E. H. Engineering Colloidal Quantum Dot Solids within and beyond the Mobility-Invariant Regime. Nat. Commun. 2014, 5, 1–7. 10.1038/ncomms4803 24801435

[ref26] Yang M.; Moroz P.; Miller E.; Porotnikov D.; Cassidy J.; Ellison C.; Medvedeva X.; Klinkova A.; Zamkov M. Energy Transport in CsPbBr_3_ Perovskite Nanocrystal Solids. ACS Photonics 2020, 7, 154–164. 10.1021/acsphotonics.9b01316.

[ref27] Protesescu L.; Yakunin S.; Bodnarchuk M. I.; Krieg F.; Caputo R.; Hendon C. H.; Yang R. X.; Walsh A.; Kovalenko M. V. Nanocrystals of Cesium Lead Halide Perovskites (CsPbX_3_, X= Cl, Br, and I): Novel Optoelectronic Materials Showing Bright Emmision with Wide Color Gamut. Nano Lett. 2015, 15, 3692–3696. 10.1021/nl5048779. 25633588 PMC4462997

[ref28] Zheng K.; Žídek K.; Abdellah M.; Messing M. E.; Al-Marri M. J.; Pullerits T. Trap States and Their Dynamics in Organometal Halide Perovskite Nanoparticles and Bulk Crystals. J. Phys. Chem. C 2016, 120, 3077–3084. 10.1021/acs.jpcc.6b00612.

[ref29] Zheng K.; Žídek K.; Abdellah M.; Chen J.; Chábera P.; Zhang W.; Al-Marri M. J.; Pullerits T. High Excitation Intensity Opens a New Trapping Channel in Organic-Inorganic Hybrid Perovskite Nanoparticles. ACS Energy Lett. 2016, 1, 1154–1161. 10.1021/acsenergylett.6b00352.

[ref30] Zhan Z.; Chen K.; Liu W.; Tang J.; Zhang H.; Leng Y.; Li R. Subwavelength-Polarized Quasi-Two- Dimensional Perovskite Single-Mode Nanolaser. ACS Nano 2021, 15, 6900–6908. 10.1021/acsnano.0c10647. 33821615

[ref31] Zheng K.; Žídek K.; Abdellah M.; Zhu N.; Chábera P.; Lenngren N.; Chi Q.; Pullerits T. Directed Energy Transfer in Films of CdSe Quantum Dots: Beyond the Point Dipole Approximation. J. Am. Chem. Soc. 2014, 136, 6259–6268. 10.1021/ja411127w. 24684141

[ref32] Neukirch A. J.; Abate I. I.; Zhou L.; Nie W.; Tsai H.; Pedesseau L.; Even J.; Crochet J. J.; Mohite A. D.; Katan C.; Tretiak S. Geometry Distortion and Small Polaron Binding Energy Changes with Ionic Substitution in Halide Perovskites. J. Phys. Chem. Lett. 2018, 9, 7130–7136. 10.1021/acs.jpclett.8b03343. 30523689

[ref33] Ai B.; Liu C.; Deng Z.; Wang J.; Han J.; Zhao X. Low Temperature Photoluminescence Properties of CsPbBr_3_ Quantum Dots Embedded in Glasses. Phys. Chem. Chem. Phys. 2017, 19, 17349–17355. 10.1039/C7CP02482G. 28650051

[ref34] Innocenzo V. D.; Grancini G.; Alcocer M. J. P.; Ram A.; Kandada S.; Stranks S. D.; Lee M. M.; Lanzani G.; Snaith H. J.; Petrozza A. Excitons versus Free Charges in Organo-Lead Tri-Halide Perovskites. Nat. Commun. 2014, 5, 3586 10.1038/ncomms4586. 24710005

[ref35] Brennan M. C.; Herr J. E.; Nguyen-beck T. S.; Zinna J.; Draguta S.; Rouvimov S.; Parkhill J.; Kuno M. Origin of the Size-Dependent Stokes Shift in CsPbBr_3_ Perovskite Nanocrystals. J. Am. Chem. Soc. 2017, 139, 12201–12208. 10.1021/jacs.7b05683. 28772067

[ref36] Peng J.; Chen Y.; Zheng K.; Pullerits T.; Liang Z. Insights into Charge Carrier Dynamics in Organo-Metal Halide Perovskites: From Neat Films to Solar Cells. Chem. Soc. Rev. 2017, 46, 5714–5729. 10.1039/C6CS00942E. 28770935

