# Supplementary material for: Charge Carrier Diffusion Dynamics in Multisized Quaternary Alkylammonium-Capped CsPbBr3 Perovskite Nanocrystal Solids
Source: ACS Appl Mater Interfaces. 2021 Sep 13;13(37):44742–50. doi: 10.1021/acsami.1c11676 (PMC8461607; doi:10.1021/acsami.1c11676)
Supplement: Supplementary file 1 — am1c11676_si_001.pdf [file am1c11676_si_001.pdf]

Supporting information for

Charge carrier diffusion dynamics in multi-sized Quaternary Alkylammonium-capped $CsPbBr_3$ perovskite nanocrystal solids

- Sol Gutiérrez Álvarez[1], Weihua Lin[2], Mohamed Abdellah[3], Jie Meng[1], Karel Zidek[4], Tonu Pullerits[2], Kaibo Zheng[1,2*]

[1] Department of Chemistry, Technical University of Denmark, DK-2800 Kongens Lyngby, Denmark.

[2] Department of Chemical Physics and NanoLund Chemical Center, Lund University P.O. Box 124, 22100 Lund, Sweden.

[3] Department of Physical Chemistry, Uppsala University, Lägerhyddsvägen 1, 752 37 Uppsala, Sweden

[4] The Research Centre for Special Optics and Optoelectronic Systems (TOPTEC), Institute of Plasma Physics, Czech Academy of Science v.v.i., Za Slovankou 1782/3, 182 00 Prague 8, Czech Republic

Calculation of $CsPbBr_3$ QDs Concentration:

The concentration of the QDs in the stock solutions (SS) were calculated using Beer´s Law, $A = \varepsilon bc$. The absorbance of the solutions was measured at 400 nm using UV-Vis spectrometer and a 1 mm path length cuvette. The molar absorptivity of the sample at 400 nm (ε_{400nm}) was calculated using the formula[3,4]:

$$\varepsilon_{400nm}\,(M^{-1}cm^{-1}) = \frac{N_A\,\sigma(cm^2)}{1000\;ln10} \qquad \text{(S1)}$$

N_A= Avogadro's constant and σ= absorption cross-section at 400 nm obtained from ref [5] for 2 sizes

Table S1. Calculation of stock solutions concentration.

Sample	σ 400 nm (cm^2)	ε400nm	OD $_{400\ nm}$ (A.U.)	Concentration (mol/L)
QD-4 SS	4.6 nm (8.97 ± 3.53) x10^{-15}	(2.3 ± 0.9) x 10^6	0.456 ± 0.004	(10 ± 4) x 10^{-7}
QD-10 SS	9.4 nm (7.87±1.29) x10^{-14}	(2.0 ± 0.3) x 10^7	0.428 ± 0.004	(1.0 ± 0.2) x 10^{-7}

Table S2 Preparation of Mixture solutions of films and R_{QD}

Volume QD-10 (µL)	Volume QD-4 (µL)	Volume Toluene (µL)	R_{QD}
0.25	100	99.75	0.03
3	100	97	0.32
5	100	95	0.53
10	100	85	1.06
15	100	80	1.58
20	100	50	2.10
50	100	0	5.09
75	100	0	7.44

The R_{QD} is calculated by

$$R_{QD} = \frac{C_{QD-10}}{C_{QD-10} + C_{QD-4}} * 100 \qquad \text{(S2)}$$

C_{QD-10} and C_{QD-4} are calculated by multiplying the volume with the stock solution concentration reported in table S1.

Distribution of QD-QD distance in the random films.

Monte Carlo simulations of random QD layers were carried out for each R_{QD}. In each case we obtain a histogram of the QD-4 to QD-10 centre-to-centre distance for the 1st, 2nd, n-closest neighbor. The histogram results are taken until the 20th neighbor and are summed and the result is fitted to a Gaussian as observed in figure 6B. The results of the center of the Gaussian peak (mean free path) and full width half maximum (FWHM) of the Gaussian fitting are presented in table S3.

Table S3 Results of mean free path

R_{QD} (%)	Mean free path (nm)	FWHM (nm)
0.3	9.68 ± 0.04	5.14 ± 0.09
0.5	9.55 ± 0.03	5.38 ± 0.07
1.1	9.58 ± 0.05	5.3 ± 0.1
1.6	10.31 ± 0.04	6.38 ± 0.1
2.1	10.24 ± 0.03	6.06 ± 0.07
5.1	11.03 ± 0.07	7.3 ± 0.2

Calculation of QD density:

QD density ($3.8x10^{17}$ cm^{-3}) of the pure QD-10 film is obtained from the Monte-Carlo simulation.

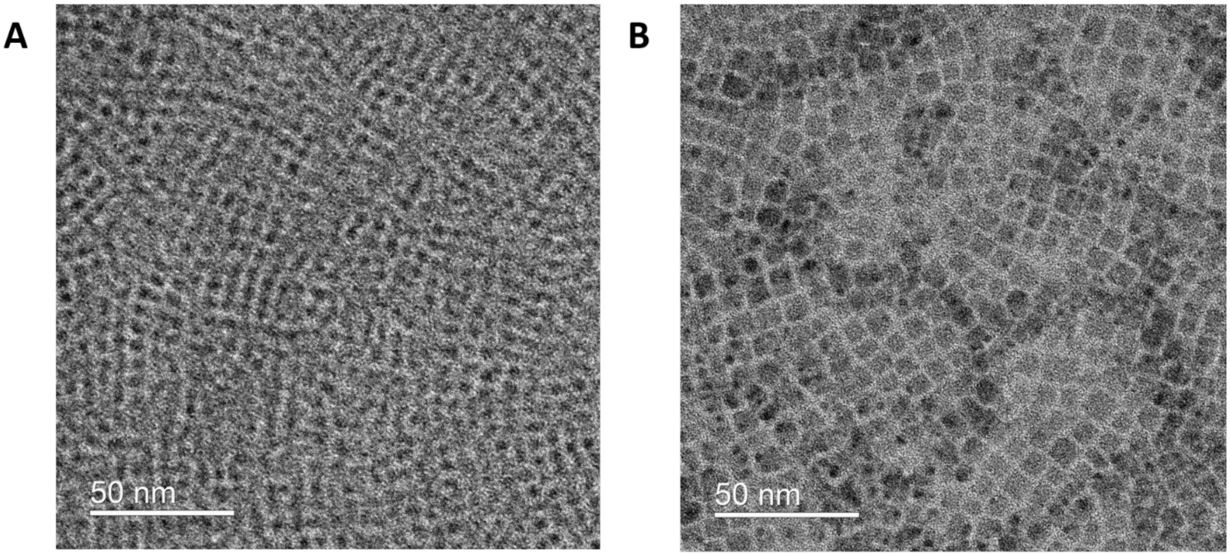

QD-4 **QD-10**

Figure S1 TEM images of A) QD-4 and B) QD-10

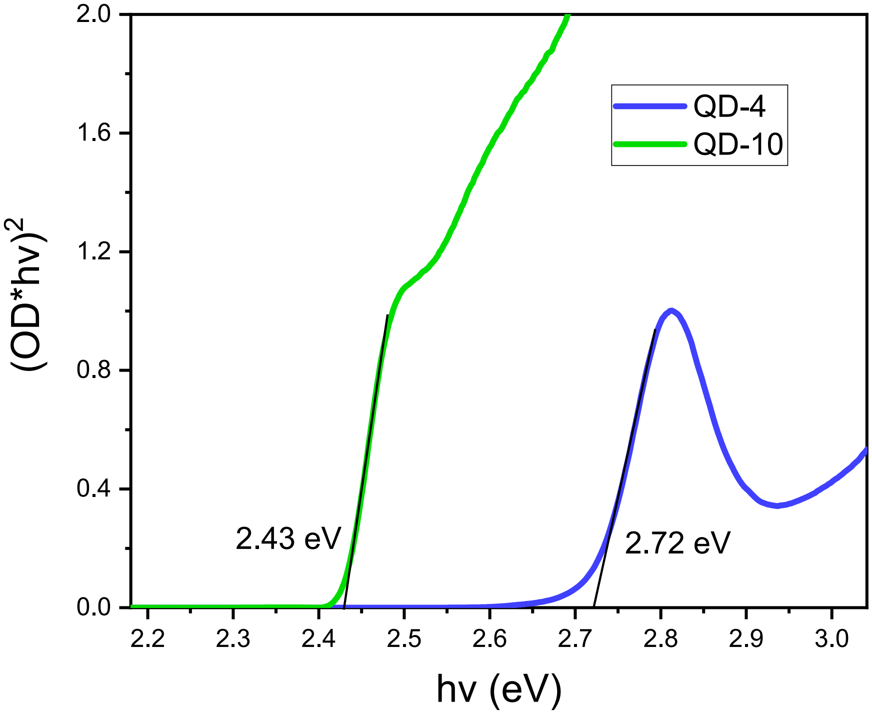

Figure S2 Tauc Plot QD-4 and QD-10

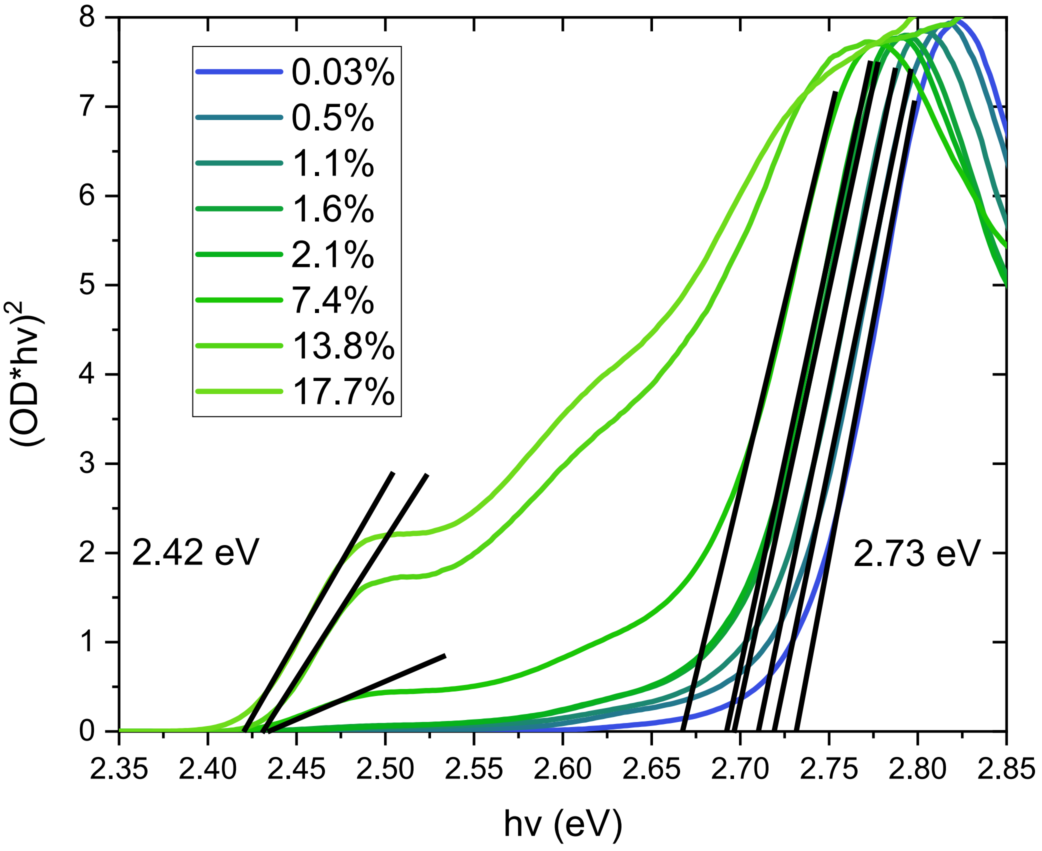

Figure S3. Tauc Plot Mixed films increasing R_{QD}.

Table S4 Summary of Band gap in mixed films with increasing R_{QD}.

R_{QD} (%)	Band Gap (eV)
0.3	2.73
0.5	2.72
1.1	2.71
1.6	2.7
2.1	2.69
7.4	2.44/ 2.66
13.8	2.43
17.7	2.42

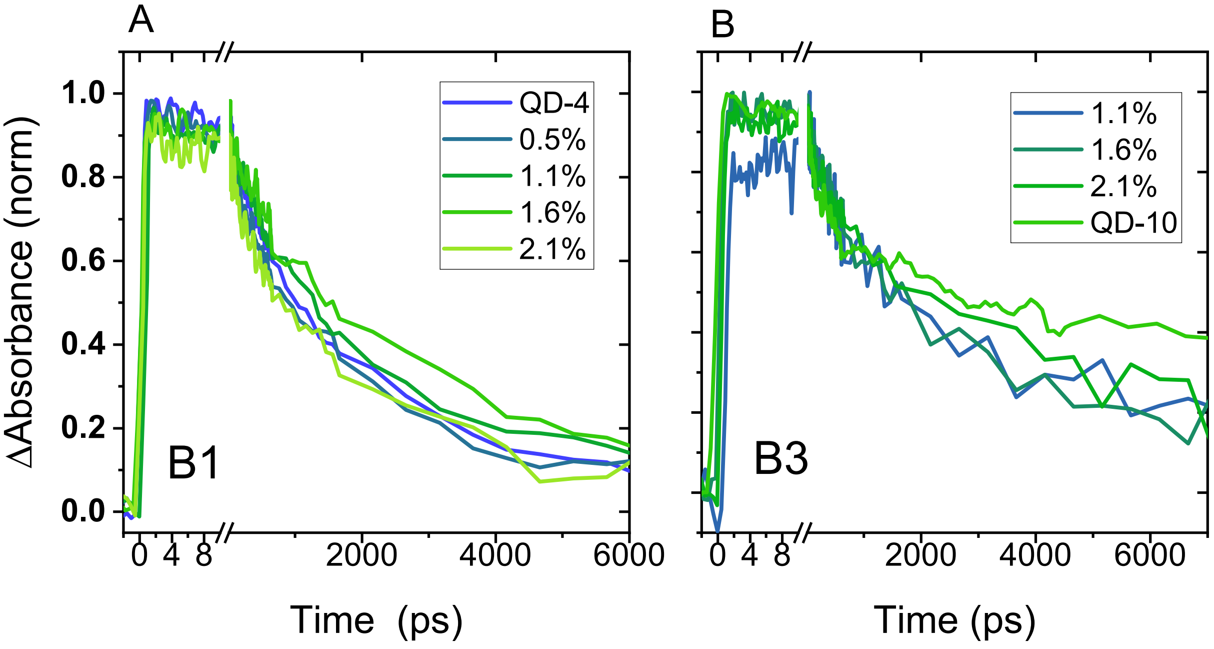

Figure S4 A)-B1 and B)-B3 kinetic traces at different percentages of $R_{QD.}$

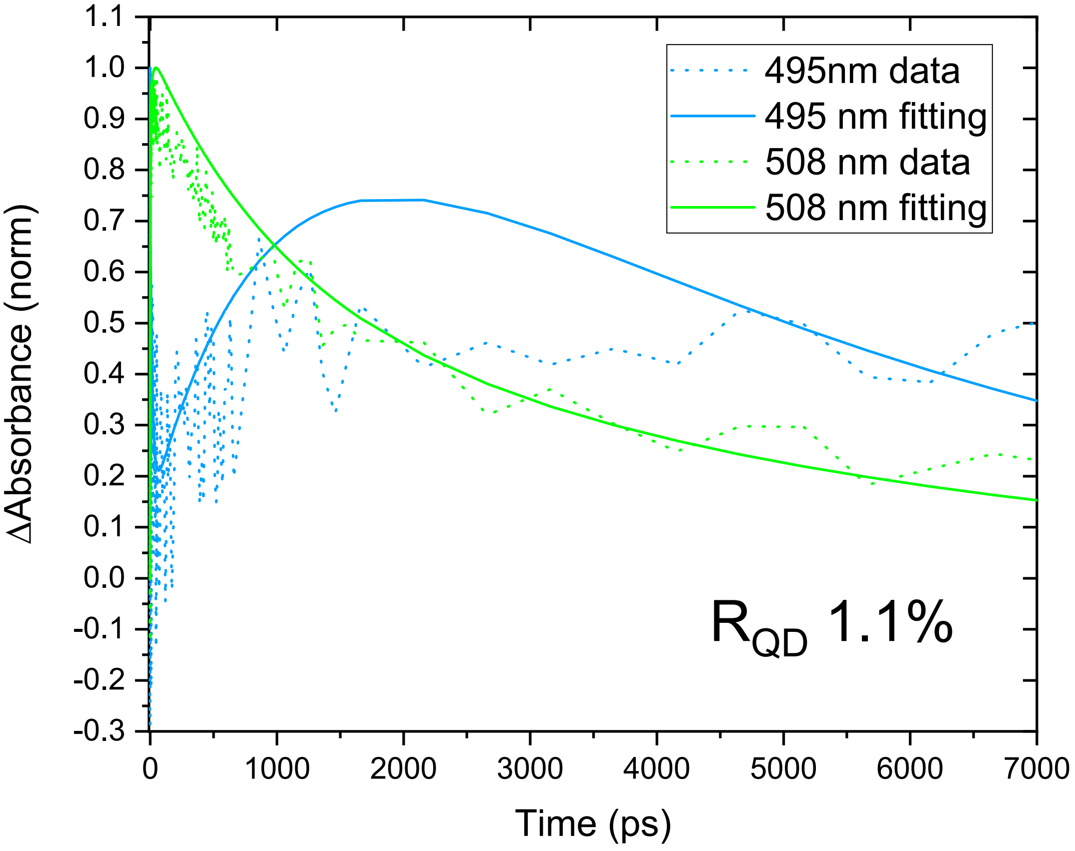

Figure S5 R_{QD} 1.1% kinetic trace and fitting at 495nm and 508nm QD-4

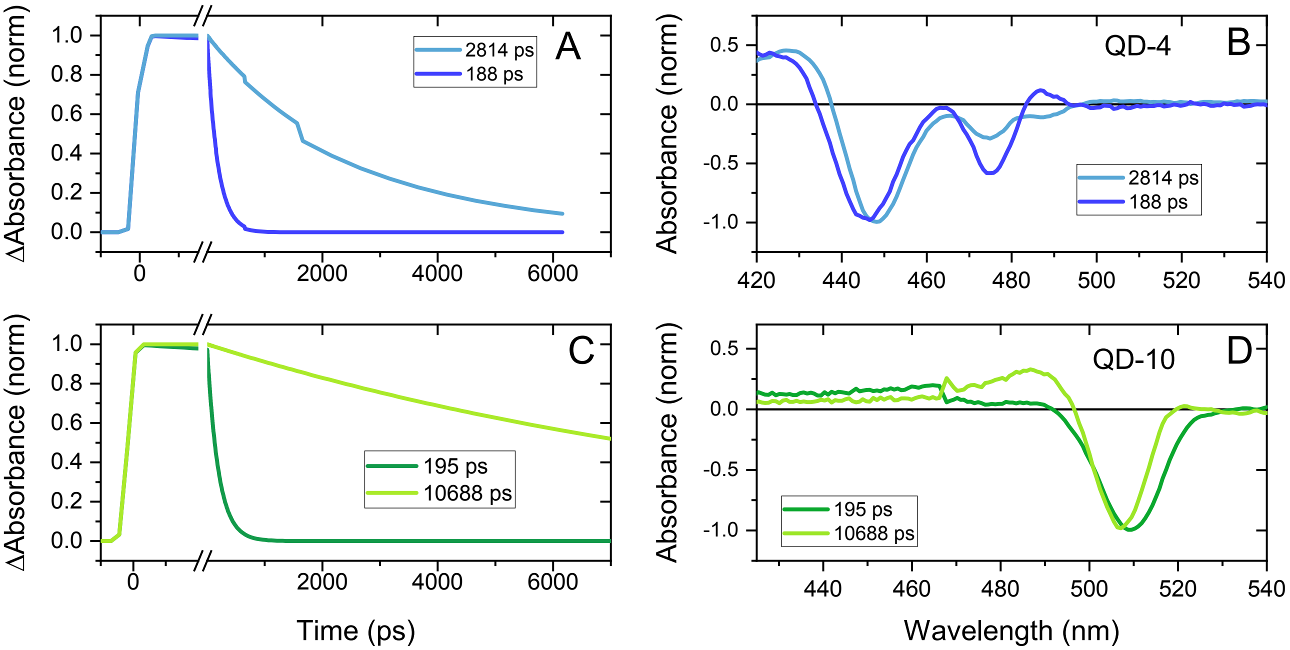

Figure S6 Associated kinetic traces of A) QD-4 and C) QD-10, and decay associated spectra of B) QD-4 and D) QD-10.

Valance band of $CsPbBr_3$

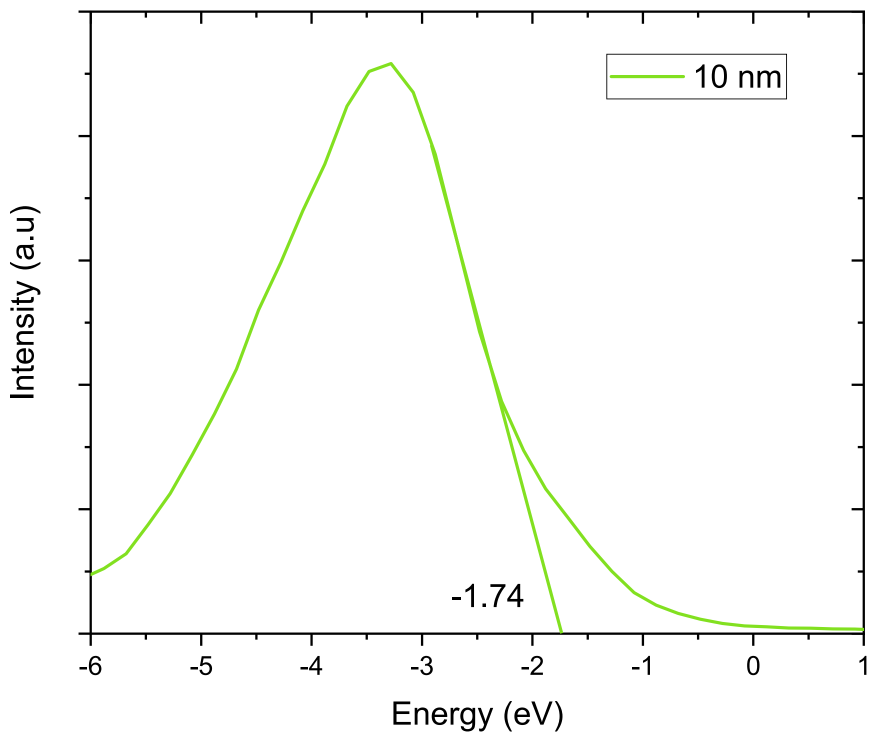

Figure S7 XPS spectra of the valance band $CsPbBr_3$

We first performed the XPS measurement of the valance band of QD-10 to confirm valance band maximum VBM position. The VBM position of QD-4 is estimated by subtracting 35 meV as previously reported in a size-dependent VB energy study.[6]

(1) Protesescu, L.; Yakunin, S.; Bodnarchuk, M. I.; Krieg, F.; Caputo, R.; Hendon, C. H.; Yang, R. X.; Walsh, A.; Kovalenko, M. V. Nanocrystals of Cesium Lead Halide Perovskites ($CsPbX_3$, X = Cl, Br, and I): Novel Optoelectronic Materials Showing Bright Emission with Wide Color Gamut. *Nano Lett.* **2015**, *15* (6), 3692–3696.

(2) Pan, J.; Quan, L. N.; Zhao, Y.; Peng, W.; Murali, B.; Sarmah, S. P.; Yuan, M.; Sinatra, L.; Alyami, N. M.; Liu, J.; Yassitepe, E.; Yang, Z.; Voznyy, O.; Comin, R.; Hedhili, M. N.; Mohammed, O. F.; Lu, Z. H.; Kim, D. H.; Sargent, E. H.; Bakr, O. M. Highly Efficient Perovskite-Quantum-Dot Light-Emitting Diodes by Surface Engineering. *Adv. Mater.* **2016**, *28* (39), 8718–8725.

(3) Brumberg, A.; Diroll, B. T.; Nedelcu, G.; Sykes, M. E.; Liu, Y.; Harvey, S. M.; Wasielewski, M. R.; Kovalenko, M. V.; Schaller, R. D. Material Dimensionality Effects on Electron Transfer Rates between $CsPbBr_3$ and CdSe Nanoparticles. *Nano Lett.* **2018**, *18* (8), 4771–4776.

(4) Jasieniak, J.; Smith, L.; Van Embden, J.; Mulvaney, P.; Califano, M. Re-Examination of the Size-Dependent Absorption Properties of CdSe Quantum Dots. *J. Phys. Chem. C* **2009**, *113* (45), 19468–19474.

(5) Chen, J.; Žídek, K.; Chábera, P.; Liu, D.; Cheng, P.; Nuuttila, L.; Al-Marri, M. J.; Lehtivuori, H.; Messing, M. E.; Han, K.; Zheng, K.; Pullerits, T. Size-And Wavelength-Dependent Two-Photon Absorption Cross-Section of $CsPbBr_3$ Perovskite Quantum Dots. *J. Phys. Chem. Lett.* **2017**, *8* (10), 2316–2321.

(6) Brennan, M. C.; Herr, J. E.; Nguyen-beck, T. S.; Zinna, J.; Draguta, S.; Rouvimov, S.; Parkhill, J.; Kuno, M. Origin of the Size-Dependent Stokes Shift in $CsPbBr_3$ Perovskite Nanocrystals. *J. Am. Chem. Soc.* **2017**, *139*, 12201–12208.
